# Get Your Facts Right: Preschoolers Systematically Extend Both Object Names and Category-Relevant Facts

**DOI:** 10.3389/fpsyg.2016.01064

**Published:** 2016-07-19

**Authors:** Amanda K. Holland, Emily Mather, Andrew Simpson, Kevin J. Riggs

**Affiliations:** ^1^Department of Psychology, Goldsmiths, University of LondonLondon, UK; ^2^School of Psychology, London Metropolitan UniversityLondon, UK; ^3^Department of Psychology, University of HullHull, UK; ^4^Department of Psychology, University of EssexColchester, UK

**Keywords:** cognitive development, language development, word learning, categorization, extension, domain general

## Abstract

There is an ongoing debate over the extent to which language development shares common processing mechanisms with other domains of learning. It is well-established that toddlers will systematically extend object labels to similarly shaped category exemplars (e.g., Markman and Hutchinson, 1984; Landau et al., 1988). However, previous research is inconclusive as to whether young children will similarly extend factual information about an object to other category members. We explicitly contrast facts varying in category relevance, and test for extension using two different tasks. Three- to four-year-olds (*N* = 61) were provided with one of three types of information about a single novel object: a category-relevant fact (‘it’s from a place called Modi’), a category-irrelevant fact (‘my uncle gave it to me’), or an object label (‘it’s called a Modi’). At test, children provided with the object name or category-relevant fact were significantly more likely to display systematic category extension than children who learnt the category-irrelevant fact. Our findings contribute to a growing body of evidence that the mechanisms responsible for word learning may be domain-general in nature.

## Introduction

Is our capacity for language the product of dedicated mental processes, or an assembly of broader cognitive mechanisms operating in unison? This question is at the heart of understanding how children learn to use and comprehend language. In the case of vocabulary development, the child requires the ability to map words to their referents. The problem, in theory, is that there is an infinite number of possible meanings for any given word (Quine, 1960). Yet, in practice, young children learn words with remarkable ease. By the age of seventeen, the average English-speaker knows more than 60,000 words (Bloom, 2000). Does this remarkable development require mental processes specific to the task of word learning?

A longstanding perspective has been that domain-specific ‘constraints and biases’ are necessary for solving the inductive difficulty of word learning (e.g., Golinkoff et al., 1994; Waxman and Booth, 2000). Others argue that domain-general processes are sufficient for the task, whether basic properties of learning, memory, and attention (e.g., Samuelson and Smith, 1998; Smith and Yu, 2008) or social pragmatic understanding (e.g., Baldwin and Tomasello, 1998). These contrasting perspectives need not be in opposition; children may be flexible using multiple cues and processes of differing specificities when word learning (Hirsh-Pasek et al., 2000; Yu and Ballard, 2007). However, the same word-learning behavior can often be explained in different ways. To give one example, toddlers display a ‘mutual exclusivity’ response where they typically select a novel, name-unknown object as the referent of a novel label, rather than a familiar, name-known object. This behavior could be the outcome of a dedicated word-learning constraint (e.g., Mervis and Bertrand, 1994), or it may involve a more domain-general attentional bias toward novel stimuli (Horst et al., 2011; Mather and Plunkett, 2012).

If word learning relies on domain-general cognitive mechanisms, then one would expect to observe parallels between the formation and retention of word mappings, and the mapping of other information. It is not just words which can be mapped to objects; other information such as associated actions, gestures, and facts about an object also require mapping. If the same behavior is evident when learning about different types of mappings, the parsimonious conclusion is that common processing mechanisms are in operation. Previous research has provided evidence that there are parallels between word learning and the mapping of actions to objects (Childers and Tomasello, 2002, 2003; Riggs et al., 2015; Dysart et al., 2016). In this paper, we focus on investigating whether there are similarities in the mapping of names and *facts* to objects.

Previous research has demonstrated that toddlers and pre-schoolers may be able to rapidly map novel nouns to objects, even with relatively brief exposure (e.g., Carey and Bartlett, 1978; Heibeck and Markman, 1987; Woodward et al., 1994; Jaswal and Markman, 2003; Holland et al., 2015), although it is less clear how well these mappings are retained over time (see Horst and Samuelson, 2008; Vlach and Sandhofer, 2012). A study by Markson and Bloom (1997) investigated whether the ‘fast-mapping’ of words to novel objects stretches to the mapping of facts. In their procedure, pre-schoolers were introduced to a novel word (e.g., “Let’s measure the *koba*”) for an unfamiliar object and also a novel fact (e.g., “We can use the thing my Uncle gave to me”) for another object. The children successfully mapped and retained both the word and the fact for the object across a retention interval of up to a month. The children performed similarly when the fact also contained a novel label (e.g., “…came from a place *called Koba*”). Markson and Bloom (1997, p. 813) concluded that the specific process of fast-mapping is not limited to words. More controversially, they further claimed to have “evidence against a dedicated system for word learning in children.”

Markson and Bloom’s case for a domain-general view of word learning was disputed by Waxman and Booth (2000). In their paper, they emphasize that word learning comprises a variety of different processes, of which fast-mapping is just one. Hence, Markson and Bloom do not have empirical evidence that *all aspects* of word learning are the outcome of domain-general processes. Waxman and Booth (2000) went on to investigate the domain-specificity of another word learning process: the extension of object names to other category members. It is well-established that toddlers and preschoolers will systematically extend a novel count noun to other members of the same object category (e.g., Markman and Hutchinson, 1984; Landau et al., 1988). Some (e.g., Markman, 1989; Booth and Waxman, 2002) argue that young children make an assumption about category membership – extending a word to other members of that category. Others argue that young children have learned to use shape-similarity as a reliable cue for count noun extension (e.g., Smith et al., 1996, 2002).

Waxman and Booth (2000) investigated whether pre-schoolers would similarly extend newly learnt facts about objects. Preschoolers were taught either a novel word, naming a novel object (“It is called a *koba*”), or a novel fact (“My Uncle gave it to me”) about a novel object. The children were then tested for categorical extension of the word or fact, either immediately after training, or after an interval of a week. Children were presented with the original target, two additional target-category members, and five other pairs of non-target exemplars. There were two free-choice tasks to test for extension: a ‘yes/no’ task and a ‘choice’ task. In the yes/no task, each object was presented and the child was asked whether the word or fact (depending on condition) applied to the object. In the choice task, all test objects were presented together and the child was asked either “Can you hand me the one that is a koba?” (Word condition) or “Can you hand me the one that my Uncle gave to me?” (Fact condition). Once a choice was made, the selected object was removed, and the question was repeated until no further selections were made. Performance on the two category extension tasks varied significantly between the word and fact conditions. Children in the word condition displayed completely systematic extension to only the target-category members in the yes/no and choice tasks, both immediately and after a delay. In contrast, children in the fact condition under-extended to target-category members and over-extended to non-target objects. Hence, there appears to be a clear pattern of categorical extension of words which does not occur for the extension of facts. Waxman and Booth (2000) argue on the basis of these results that there are different processes involved in the extension of words and facts. Thus, word learning may not involve purely domain-general processes as proposed by Markson and Bloom (1997). This differential category extension of words and facts has also been reported for children as young as 2.5 years of age (Behrend et al., 2001).

However, there is an important difference between the words and facts used in this previous research. The novel words are count nouns, as indicated by their grammatical form, e.g., ‘This is *a* koba.’ Importantly, a count noun applies not just to the originally labeled exemplar, but also to all other members of the relevant category. However, it is far from clear that the novel facts tested share this property of applying to all members of the labeled category. The fact ‘My Uncle gave it to me,’ used by Waxman and Booth (2000), would normally be interpreted as applying only to that particular item, rather than as a fact which applies to other category members (see also Childers and Tomasello, 2003). Moreover, the pragmatic context (interpreting an unfamiliar experimenter) may leave children uncertain over whether or not to extend the new fact, creating inconsistent or *ad hoc* patterns of extension. A similar argument can be made about the facts used by Behrend et al. (2001), such as ‘the thing that fell in the sink.’ Therefore, differences between the facts and words employed in this research may simply reflect the kind of facts used, rather than a fundamental difference between facts and words. A fairer comparison with count nouns requires facts which are more readily interpreted as relevant to category membership, i.e., facts which do not concern unique or accidental properties of a specific item.

Other studies have distinguished between facts which apply only to individual objects and facts which apply to a category of objects. In an experiment reported by Diesendruck and Bloom (2003), three-year-olds were provided with either category-relevant facts (e.g., ‘it is used in the kitchen’) or category-irrelevant facts (e.g., ‘I got this for my birthday’) about novel objects. The children were then presented with a *forced-choice* extension test. Children had a choice to extend the new fact to another object which was either a shape match, a color match, or a material match to the original referent. Children presented with category-relevant facts were significantly more likely to make shape-based extensions, than children provided with category-irrelevant facts. However, the bias to make a shape match was significantly above chance for children in both conditions. At first sight, the children appear to be extending facts which should be restricted to individual objects. Yet, the use of a ‘forced-choice’ procedure means that the children are *required* to extend the fact to one of the three objects. As suggested by Diesendruck and Bloom (2003), children in this condition may have defaulted to an ‘extend by shape’ strategy, given that shape is otherwise a reliable cue for extension. Moreover, the same argument can be made for the category-relevant facts – did the children really want to make a category extension, or was it merely an artifact of the testing procedure?

Finally, Deák and Toney (2013) observed that 4- to 5-year-olds extend novel facts, in apparent contradiction to the findings of Waxman and Booth (2000). Deák and Toney (2013) state that their facts are ‘neutral’ with respect to being category-relevant. However, some of the facts used are arguably specific to unique exemplars (e.g., ‘my sister gave this to me,’ ‘I keep this on my desk’), whereas others are more likely to apply to object categories (e.g., ‘this is from Japan’). Deák and Toney (2013) do not report data separately for each fact; hence it is unclear whether or not the children were discriminating between them on the basis of category relevance.

In sum, given the disparate findings across studies, we aim to clarify whether or not facts are systematically extended to other members of a category, and furthermore, what *kinds* of facts might be extended. Evidence for the categorical extension of both facts and nouns would provide further support for the domain-generality of word learning. Presently, there is ambiguity in the literature over how facts are classified with regards to category relevance. In the experiment reported below, we clearly distinguish between facts varying in category relevance, similar to Diesendruck and Bloom (2003). This manipulation allows us to test for patterns of extension in the predicted direction. Crucially, children’s *discrimination* of different types of facts would provide more convincing evidence that category-relevant facts are truly classified and extended as such, rather than the outcome of an *ad hoc* strategy. We additionally contrast two kinds of facts with count nouns. Unlike Diesendruck and Bloom (2003), we use tests of category extension similar to Waxman and Booth (2000) – a free choice task, and a yes/no task. This testing procedure does not force children to extend the fact or word to at least one item. Therefore, all extension we do observe is a true reflection of children’s preferences. We hypothesized that there would be no significant difference in the extension of nouns and category-relevant facts, but both would be significantly greater than the extension of category-irrelevant facts.

## Materials and Methods

### Participants

A total of 73 three- to four-year-olds originally participated and were tested for comprehension. Of these, 61 children correctly identified the original referent of the noun or fact, and went on to be tested for extension. In the Object Label condition, there were 19 children (9 male, 10 female) with a mean age of 3.93 years (*range* = 3.34–4.94). In the Category-irrelevant condition, there were 21 children (9 male, 12 female) with a mean age of 3.92 years (*range* = 3.21–4.94). In the Category-relevant condition, there were 21 children (6 male, 15 female) with a mean age of 3.84 years (*range* = 3.20–4.52). There were no significant differences in age, gender or vocabulary across conditions for tests of comprehension or extension (all *p*s > 0.4).

### Stimuli

#### Exposure Array and Comprehension Test Array

**FIGURE 1:**
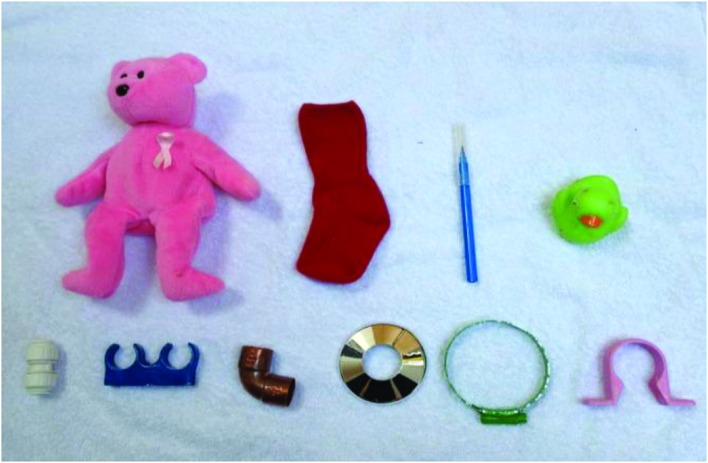
**Exposure Array and Comprehension Test Array.** *From top, left to right:* pink teddy, red sock, blue pen, green duck, white connector, blue double pipe clip, copper elbow, chrome pipe collar, green hose clip and pink pipe clip.

The Exposure Array, presented to the children during the Exposure Session, comprised ten objects – six novel and four familiar (See **Figure 1**). Children’s’ comprehension of the link between the object and the novel object label or fact was tested using the same array. The novel objects were sourced from a large DIY store and will be referred to as a connector, double pipe clip, elbow, pipe collar, hose clip, and pipe clip. The four familiar objects were a pink teddy, a red sock, a blue pen, and a green duck. During the Exposure Session the objects were placed upon a plain white towel.

#### Extension Array

**FIGURE 2:**
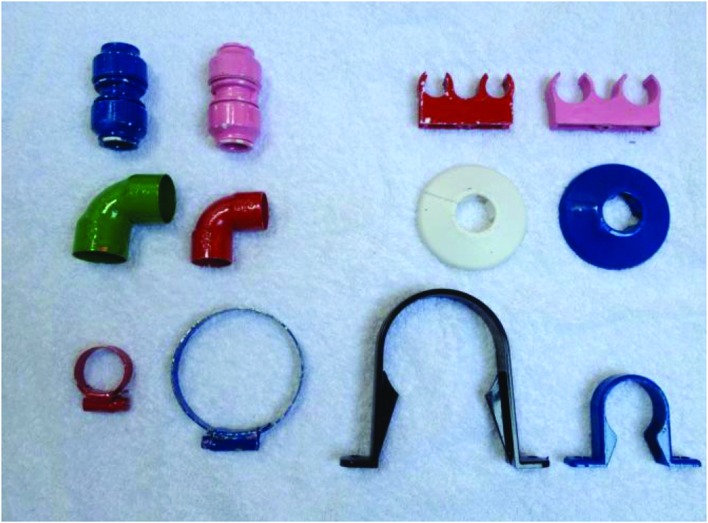
**Extension Array.** *From top, left to right (six pairs):* blue connector and pink connector; red double pipe clip and pink double pipe clip; green elbow and red elbow; white pipe collar and blue pipe collar; red hose clip and blue hose clip; black pipe clip and blue pipe clip.

The Extension Array (see **Figure 2**) presented to children during the Extension Test comprised 12 novel objects. There were six pairs of two exemplars of each of the novel objects from the exposure array. These exemplars shared the same shape with the original exposure objects, but differed in color and/or size. The extension array did not include the original target object (cf. Waxman and Booth, 2000) to ensure an equal number of exemplars for each object category. The presence of the original target could otherwise bias selection of the target category.

### Design

This study consisted of a between-participants experimental design. A single independent variable of *Information Type* was manipulated to vary the novel information provided about the novel object. There were three Information Type conditions: *Object Label*, *Category-irrelevant*, and *Category-relevant*. All children were tested immediately after the exposure session. There were two dependent variables: *Comprehension Accuracy* and *Extension Accuracy*.

### Procedure

Ethical approval was granted by the ethics committee of London Metropolitan University. Informed consent was obtained from parents and the head of the nursery school. The procedure was based on Waxman and Booth (2000). All children initially underwent a fast mapping task, where each child was introduced to a novel count noun or novel fact in the exposure session. Their comprehension and extension of this novel word or fact was assessed in the testing session that immediately followed the exposure session.

#### Exposure Session

Each child sat down at a table where a white towel was laid out. They were presented with a transparent box containing six novel objects and four familiar objects (See **Figure 1**). They were asked to get all ten objects out of the box and put them on the table. This ensured that the children touched and looked at each object, for roughly equivalent amounts of time. Following this brief introduction to the objects, the experimenter started the main task. The experimenter said, “Look. Here I have a towel” and moved the ten objects to the side of the towel. Then the experimenter said, “I want to put all of these things onto my towel so that it makes a fun picture. Can you show me where to put them so it looks really good? We’ll do it one at a time so, we don’t miss any out.” The experimenter picked up one of the ten objects and said, “Let’s start with this one.” After the child had placed the object somewhere on the towel, the experimenter praised the participant and picked up another object asking, “Where would you put this one?” The experimenter continued this process with each of the objects asking, “And how about this one?” waiting for the child to place the object before going on to the next one. Objects were chosen at random except that the target object was never first or last. For all conditions, each of the novel objects served as the target object in rotation across participants.

The experimenter introduced some new information about the target object. In the Object Label condition, the experimenter said “This is really special – it’s called a *modi* – where do you want to put this one?” In the Category-irrelevant condition, the experimenter said “This is really special – *my uncle gave it to me* – where do you want to put this one?” In the Category-relevant condition, the experimenter said “This is really special – *it’s from a place called Modi* – where do you want to put this one?” Once all the objects had been placed on the towel the experimenter said, “That’s brilliant, thank you. I think that looks really great. What do you think? Are you happy with it?” and children were allowed to change the position of any of the objects if desired.

#### Comprehension Test

All children took part in the Comprehension Test session directly following the Exposure Session. Depending upon when the target object was presented during the exposure session, the child experienced a gap between exposure to the word or fact mapping and subsequent comprehension testing that ranged from approximately 30 s to 2 min.

Referring to the array of ten objects the experimenter said to the child, “We’re going to put these away.” The experimenter then asked one of three questions: “But just before we do, can you show me which one is called a modi?” (Object Label condition) “….can you show me which one my uncle gave to me?” (Category-irrelevant condition) or “….can you show me which one comes from a place called Modi?” (Category-relevant condition). Their answer confirmed whether they had retained the word or fact mapping.

The experimenter ended the Comprehension Test by saying, “Can you help me by putting the things away now? They all go back in the box.” For children who did not choose the target object, this was the end of their participation in the experiment. Children who answered the comprehension test correctly were tested for extension of the newly learned word or fact to additional objects from target and non-target categories.

#### Extension Test

Once the Exposure and Comprehension Test Array had been tidied away, the experimenter opened the transparent box containing the Extension Array (See **Figure 2**) and placed the twelve objects randomly on the table in front of the participant. All children underwent two extension tests similar to those used by Waxman and Booth (2000). There was a Yes/No task and a Choice task. To control for order effects the presentation of these tasks was counterbalanced.

##### Yes/No task

The experimenter pointed to each object in turn, in a random order, and asked, “Is this one a modi?” (Object Label condition), “Is this one my uncle gave to me?” (Category-irrelevant condition) or “Is this one from a place called Modi?” (Category-relevant condition).

##### Choice task

The experimenter asked, “Can you see anything here that’s called a modi?” (Object Label condition), “Can you see anything here that my uncle gave me?” (Category-irrelevant condition) or “Can you see anything here that comes from a place called Modi?” (Category-relevant condition). After the participant’s initial selection, the experimenter removed that object and prompted the child for additional selections. For example, in the Object Label condition, the experimenter said, “Are there any other ones that are modis?” As in Waxman and Booth (2000), the experimenter repeated this choice question until the child did not select any other objects.

#### Vocabulary Test

Children’s vocabulary was tested approximately 1 week after the comprehension and extension testing using The British Picture Vocabulary Scale: Third Edition (BPVSIII) scale (Dunn et al., 2009).

### Scoring

For all conditions, selecting target objects earned a positive score, selecting a non-target object earned a negative score, whilst non-selection scored zero points across both extension tests. Therefore, selecting *only* the target objects earned maximum points. Scores for selecting target objects were weighted, as there was a far larger proportion of non-target (10) to target objects (2) – a ratio of 5:1. If random performance is what participants would pick with their eyes shut, irrespective of how many picks they make, they are five times more likely to pick a foil than a target.

#### Yes/No

‘Yes’ responses received a score of +5 for the target exemplars and -1 for the non-target category objects. ‘No’ responses received a score of 0. This produced a score ranging from +10 to -10 for each child.

#### Choice

Selecting objects from the target category received a score of +5, selection of the non-target objects received a score of -1 and objects that were not selected received a score of 0. This produced a score ranging from +10 to -10 for each child.

## Results

### Comprehension Accuracy

Of the 73 participants, 61 answered the comprehension question correctly by choosing the target object. As expected when tested immediately, a high proportion (79–88%) of the children could demonstrate their understanding of the mapping between the target object and the novel word or fact (See **Table 1**). There was not a significant difference in comprehension accuracy across conditions, χ^2^ = 0.664, *p* = 0.798 (Fisher’s Exact Test). Binomial tests showed that performance was greater than expected by chance (1 in 6) in all three Information Type conditions (*p* < 0.001).

**Table 1:** Comprehension Test: accuracy by condition.

Object selected	Object label	Category-irrelevant	Category-relevant	Total
Target	19	21	21	61
Non-target	5	3	4	12
Percentage of participants selecting the target	79%	88%	84%	84%

### Extension Accuracy

The 61 children who correctly chose the target in the comprehension test, also completed the extension test. Preliminary analyses of differences across the Information Type conditions revealed very similar patterns of data for the Yes/No and Choice tasks. These similarities were confirmed by significant correlations between the tasks by condition (all *rs* ≥ 0.45, *ps* ≤ 0.031). For brevity, we therefore report the analysis of scores aggregated across the two tasks. The weighted extension scores for each test were added together to provide a total weighted extension score for each participant, which could range in integers from minus 20 (exclusively selecting non-target exemplars) through zero to a maximum of plus 20 (exclusively selecting target exemplars). This aggregate score provides a complete picture of how the participant performed over both tests and does not obscure any inconsistent response patterns between the two tests.

**FIGURE 3:**
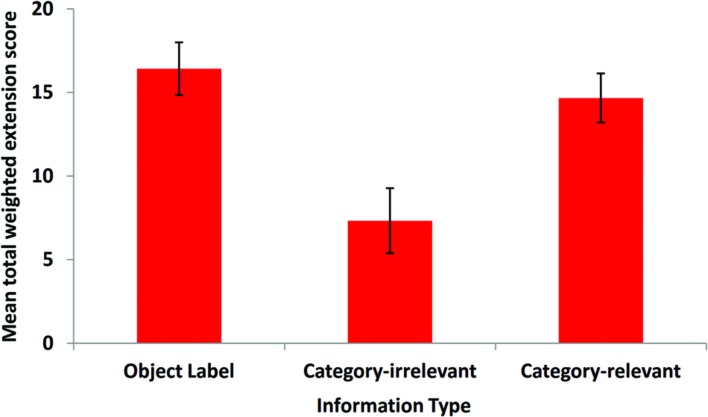
**Mean extension test scores by Condition (error bars are ± 1 SE)**.

The total weighted extension scores are presented in **Figure 3** The children in the Object Label condition (*M* = 16.42, *SD* = 6.68) and the Category-relevant condition (*M* = 14.67, *SD* = 6.73) tended to choose objects from the target category at a greater rate than children in the Category-irrelevant condition (*M* = 7.33, *SD* = 8.91). A One-Way ANOVA revealed a significant effect of Information Type on children’s extension of novel words and facts, *F*Welch (2,58) = 6.938, *p* = 0.003 (Welch’s F statistic for unequal variances). *Post hoc* multiple comparison tests (Games-Howell correction) revealed a significant difference between the Object Label and the Category-irrelevant conditions (*p* = 0.002) and, crucially, a significant difference between the Category-relevant and Category-irrelevant conditions (*p* = 0.013). There was no significant difference between the Category-relevant and Object Label conditions (*p* = 0.696).

### Individual Extension Patterns

The above analyses have revealed lower scores for children in the Category-irrelevant condition than for children in either the Object Label or Category-relevant conditions. To better understand why this difference occurred, every child’s performance was classified into one of three primary response patterns for both extension tasks. A ‘target category only’ extension pattern described participants who selected both exemplars of the target category, but no other test objects. An ‘extend to all’ extension pattern described participants who selected at least 11 of the 12 test objects. An ‘inconsistent’ extension pattern described participants who selected objects from the target and non-target categories of objects. Only two participants selected no test objects at all within a task, and neither did this for both tasks. Three participants’ did not select the target category but their selection was limited to a single non-target category. Only one of these three participants replicated this pattern of responding across both the Yes/No and Choice tasks. The remaining selection patterns were seemingly random. Hence, all these extension patterns were summarized as ‘inconsistent.’

Children’s extension patterns for each extension task are presented in **Table 2** The Object Label and Category-relevant conditions exhibited similar extension patterns – most children (67–79%) extended only to the target category in both the Yes/No and the Choice tasks. In contrast, less than 40% of children in the Category-irrelevant condition extended only to the target category in either task. The remaining children in the Category-irrelevant condition were split fairly evenly between the ‘Extend to All’ and the ‘Inconsistent’ extension patterns. A 3 × 3 χ^2^ test on the Yes/No data demonstrates a significant relationship between condition and extension pattern, χ^2^ = 15.346, *p* = 0.003 (Fisher’s Exact Test). The Choice data provide similar results, χ^2^ = 10.574, *p* = 0.026 (Fisher’s Exact Test).

**Table 2:** Extension Patterns by Information Type: the number and percentage of participants.

Information type	YES/NO			CHOICE		
	Target category only	Extend to all	Inconsistent	Target category only	Extend to all	Inconsistent
Object label	14	0	5	16	1	2
	74%	0%	26%	84%	5%	11%
Category-irrelevant	4	6	11	8	7	6
	19%	29%	52%	38%	33%	29%
Category-relevant	12	4	5	16	3	2
	57%	19%	24%	76%	14%	10%

Collapsing the extension patterns into two categories: ‘Target Category Only’ and ‘Other’ allows *post hoc* multiple comparisons using 2 × 2 χ^2^ tests to explore differences between pairs of conditions. The adjusted critical *p*-value of 0.025 reflects the fact that the data for each condition in the *post hoc* comparisons has been analyzed twice. There was a significant difference between the children’s extension pattern in the Category-irrelevant and the Category-relevant conditions in the Yes/No test, χ^2^ = 6.462, *p* = 0.011 and in the Choice test, χ^2^ = 6.222, *p* = 0.013. In contrast, a comparison of Object Label and Category-relevant for both the Yes/No task (χ^2^(1) = 1.200, *p* = 0.273) and the Choice task (Fisher’s Exact Test, *p* = 0.698) reveals that children do not extend general facts in a significantly different way from object labels.

## Discussion

The current experiment compared children’s extension of an object label and two different kinds of facts. The specific fact was relevant to an individual object (“My uncle gave this to me”), whereas the general fact was relevant to the category from which the object came (“It comes from a place called Modi”). Following Waxman and Booth (2000), we used extension tasks which allowed children to freely decide whether or not to extend the word or fact (cf. Diesendruck and Bloom, 2003). It was found that children’s extension pattern varied as a function of condition. Children in the Category-relevant and Object Label conditions displayed similar response patterns of exclusively selecting members of the target object category. In contrast, children in the Category-irrelevant condition were more likely to extend the specific fact to non-target category objects than children in either the Category-relevant or Object Label conditions. It would appear that if the fact is category-relevant rather than object-specific, children will systematically extend the fact to appropriate same-shaped objects. These results strongly suggest that children can extend a fact to other same-category items just like they do with words.

Our study is not the first to provide evidence of preschoolers’ extension of facts to same-category exemplars. However, we provide a more stringent demonstration that young children are capable of identifying a novel fact as category-relevant, and to spontaneously extend the fact to other category members. In contrast to Diesendruck and Bloom (2003), the use of a free choice procedure means that the children were not forced to select an object during the extension test. Furthermore, we explicitly compare children’s responses to facts varying in category relevance. Thus, we have evidence that the extension of facts is actually sensitive to category relevance, rather than occurring as an indiscriminate, *ad hoc* strategy. Our findings extend the work of Diesendruck and Bloom (2003) and Deák and Toney (2013) and demonstrate that young children’s readiness to extend category-relevant facts stands up to a more robust test of extension.

One issue, which remains, concerns the extension pattern of children in the Category-irrelevant condition. It is less clear why children in the Category-irrelevant condition chose to extend the fact to some (or all) of the target and non-target exemplars, when it arguably applies only to the originally designated object. Given the use of a free choice task, one might have expected the children not to have extended the fact at all. However, the experiment may have nonetheless placed pragmatic ‘pressure’ on the children’s responses. For example, children may have thought that the experimenter would not ask the question if the answer was no. Moreover, with such a large array of test objects available, children might think it odd for the fact not to apply to at least some of the objects present. Alternatively, the children may have struggled to clearly classify the fact as either category-relevant or category-irrelevant, resulting in less coherent extension patterns both within and across participants. Children in the Category-irrelevant condition exhibited a larger number of inconsistent selection patterns. Our experiment was not designed to systematically investigate which foils were selected. This would be an interesting avenue for future research. An error analysis may provide some insight into children’s extension choices for category-irrelevant facts.

Waxman and Booth (2000, 2001) have argued that the extension of facts may not display the same characteristics as the extension of nouns. They argue that while the appropriate extension pattern for nouns can largely be determined by grammatical form, the extension profile for facts is much less clear and often depends on broader world knowledge. While this might be true, Waxman and Booth point to differences between the nature of words and facts themselves, rather than differences in the mechanisms underlying extension. Arguably, a fact about the geographic origin of an object may not serve to define category membership in the same manner as an object label. Yet, we have shown that when a fact can be interpreted as having relevance to a category, it will be systematically extended to other category exemplars. Importantly, the children had no prior experience of the novel fact and how it is extended. Thus, the spontaneous and systematic extension of a novel fact suggests a general mechanism for extension, rather than case-by-case learning. Further research will need to establish whether, under suitable experimental conditions, facts which denote specific objects will be strictly restricted to the original referent.

A final caveat is that the fact introduced to children in the Category-relevant condition was “It comes from a place called Modi” which contains a novel non-word. Perhaps children are extending this fact, not because it’s category-relevant, but because they associate the *novel word* with the target object. They may then extend this novel word, rather than the fact, to other similar-shaped objects. However, this interpretation is unlikely for two main reasons. First, other researchers have shown that children will not extend category-irrelevant facts when they do contain a novel word (Behrend et al., 2001) and, vice versa, children will extend category-relevant facts when they don’t contain a novel word (Diesendruck and Bloom, 2003). Second, the category-relevant fact used in the present study introduces a novel *proper noun*, and pre-schoolers have been shown not to extend proper nouns (see Hall, 1999). So, if children in this experiment had linked the novel word rather than the fact with the object, they would have been more likely to extend the fact at significantly less than chance levels – neither to the target or non-target category objects. This was not the case.

So it would appear that children treat the extension of novel category-relevant facts and novel object labels that they have fast mapped in a very similar way. This does not necessarily mean that the same mechanism in the brain is used for extension of linguistic facts and words. However, arguing that there are two separate systems (for words and facts) determining whether a piece of information applies to an individual or category seems a less likely explanation, and certainly a more complex one. Furthermore, our findings are consistent with other studies demonstrating parallels between word learning and the mapping and extension of *other* types of information to objects (Childers and Tomasello, 2002, 2003; Riggs et al., 2015). Thus, contrary to the view of Waxman and Booth (2000), the extension of words appears to be part of a more domain-general mechanism. The evidence here lends support to Bloom's (2000) theory that word learning is domain-general, drawing upon a variety of general cognitive processes in a unique way to form and retain word meanings.

## Author Contributions

AH conceived and designed the experiment with input from AS and KR. AH collected and analyzed the data. All authors contributed to the theoretical interpretation of the data. AH and EM drafted the manuscript with input from AS and KR. All authors approved the final version of the manuscript for publication.

## Conflict of Interest Statement

The authors declare that the research was conducted in the absence of any commercial or financial relationships that could be construed as a potential conflict of interest.
